# Long Term Exposure to Virgin and Recycled LDPE Microplastics Induced Minor Effects in the Freshwater and Terrestrial Crustaceans *Daphnia magna* and *Porcellio scaber*

**DOI:** 10.3390/polym13050771

**Published:** 2021-03-02

**Authors:** Anita Jemec Kokalj, Andraž Dolar, Jelizaveta Titova, Meeri Visnapuu, Luka Škrlep, Damjana Drobne, Heiki Vija, Vambola Kisand, Margit Heinlaan

**Affiliations:** 1 Biotechnical Faculty, University of Ljubljana, Večna pot 111, 1000 Ljubljana, Slovenia; anita.jemec@bf.uni-lj.si (A.J.K.); Andraz.Dolar@bf.uni-lj.si (A.D.); damjana.drobne@bf.uni-lj.si (D.D.); 2 Laboratory of Environmental Toxicology, National Institute of Chemical Physics and Biophysics, Akadeemia tee 23, 12618 Tallinn, Estonia; jelizaveta.titova@kbfi.ee (J.T.); heiki.vija@kbfi.ee (H.V.); 3 Institute of Physics, University of Tartu, W. Ostwaldi Str 1, 50411 Tartu, Estonia; meeri.visnapuu@ut.ee (M.V.); vambola.kisand@ut.ee (V.K.); 4 Slovenian National Building and Civil Engineering Institute, Dimičeva ulica 12, 1000 Ljubljana, Slovenia; luka.skrlep@zag.si

**Keywords:** microfragments, chronic, OECD211, water flea, isopod, woodlice, low density polyethylene, additive

## Abstract

The effects of microplastics (MP) are extensively studied, yet hazard data from long-term exposure studies are scarce. Moreover, for sustainable circular use in the future, knowledge on the biological impact of recycled plastics is essential. The aim of this study was to provide long-term toxicity data of virgin vs recycled (mechanical recycling) low density polyethylene (LDPE) for two commonly used ecotoxicity models, the freshwater crustacean *Daphnia magna* and the terrestrial crustacean *Porcellio scaber*. LDPE MP was tested as fragments of 39.8 ± 8.82 µm (virgin) and 205 ± 144 µm (recycled) at chronic exposure levels of 1–100 mg LDPE/L (*D. magna*) and 0.2–15 g LDPE/kg soil (*P. scaber*). Mortality, reproduction, body length, total lipid content, feeding and immune response were evaluated. With the exception of very low inconsistent offspring mortality at 10 mg/L and 100 mg/L of recycled LDPE, no MP exposure-related adverse effects were recorded for *D. magna*. For *P. scaber*, increased feeding on non-contaminated leaves was observed for virgin LDPE at 5 g/kg and 15 g/kg. In addition, both LDPE induced a slight immune response at 5 g/kg and 15 g/kg with more parameters altered for virgin LDPE. Our results indicated different sublethal responses upon exposure to recycled compared to virgin LDPE MP.

## 1. Introduction

The exponentially increasing use of plastics in all areas of human activity has resulted in greater amounts of plastic waste that is often mismanaged and has been considered a persistent pollutant [1] and a hazardous waste [2], reflecting the severity and extent of the plastic pollution problem. In time, the share of smaller size fractions in the overall plastic waste continues to increase due to material fragmentation and the long-term hazards of smaller fractions (e.g. microplastics ≤5 mm, MP) should thus be better understood. Adhering to the European Plastics Strategy [3], one of the aims of the European Green Deal [4] is achieving reusability/recyclability of packaging by 2030. In Europe, low density polyethylene (LDPE) is the second most used plastic polymer after polypropylene (PP) and while it dominates the packaging/film industry yet it is less recycled compared to PP and high-density polyethylene (HDPE) packaging [5]. Intensive use of polyethylene (PE) is also reflected in the MP contamination [6], where it is among the dominant polymer types found in drinking water [7], fresh water [8,9] marine [10,11,12] and terrestrial environments [13,14] and outdoor air [15,16].

Based on monomer hazard classification, LDPE has been ranked among the least hazardous of the virgin plastics [17], but this hazard ranking may not be true for recycled plastics. Recycled plastics contain a multitude of (un)intentionally added chemical additives/contaminants (e.g. pesticide residues, pigments, flame retardants) [18,19] identification of which alone is challenging [20] and establishing polymer-based toxicological signature more so [21].

To date, hazard research on plastic pollution has remained rather focused on the marine environment [22,23], yet the mismanaged plastic reaches the oceans mostly because of the riverine transport of mismanaged plastic waste from land-based sources [24,25]. There is substantial evidence that terrestrial ecosystems, especially agricultural soils, are contaminated with MP [26,27,28], mainly via deposition of sewage sludge as fertiliser [27], irrigation and aerial deposition [29] and to large extent via plastic mulching, which has become a globally applied agricultural practice [30,31]. PE and in particular, LDPE, are by far the most commonly used materials in agricultural mulch production [30,32]. In soils that had been continuously mulched with plastic film for 30 years, up to 40 mg/kg (0.004% *w*/*w*) [33] of PE MP was recorded. In freshwater systems, wastewater treatment plants’ inadequate waste management and industrial practices [8] have all been considered to be among the main sources of MP pollution. MP concentrations in freshwater have been shown to span orders of magnitude from 0 in the Laurentian Great Lakes in the US [34] to 172,000 to 519,000 MP fibers/m^3^ and 10–223 MP fragments/m^3^ in the Saigon River in Vietnam [8].

LDPE MP exposure has been shown to affect both terrestrial and aquatic invertebrates. Reduced survival and growth rate of earthworm *Lumbricus terrestris* [35], decreased reproduction and avoidance behaviour of springtail *Folsomia candida* [36], morphological damage and antioxidant response of earthworm *Eisenia fetida* [37], changes in gut bacterial community structure and diversity of coleoptera *Tenebrio molitor* larvae [38] and *F. candida* [36] have been recorded upon exposure to LDPE MP. In contrast, there are also terrestrial studies with no observed toxicity upon long-term LDPE MP exposure [39,40]. The studies have used virgin commercially supplied LDPE, with the exception of [40] who used LDPE milled from used consumer products (plastic bags). In fresh water, virgin LDPE has been shown to induce only minor changes in macromolecular biomarkers [41], ranking among the least hazardous polymers [17]. Likewise, no morphological and molecular changes have been recorded in *Daphnia magna* for other polymers (e.g. ethylene acrylic acid copolymer) [42] and their mixtures incl. those from among the most hazardous polymers [43]. Again, these studies have been conducted on virgin plastics with a few exceptions in studied MP from consumer products. Xu et al. [44] showed exposure to post-consumer mixed plastic leachate containing differently sized MP did not affect survival of *D. magna*, but increased growth and reproduction that was attributed to hormesis and the potential role of plastic additives. Schür et al. [45] showed irregular PS MP to affect *D. magna* life-history endpoints differently from natural kaolin particles. The only study on the effects of recycled plastics (PE) MP for *D. magna* reported inhibited egestion and immobilization upon exposure to irregular PE fragments compared to same-sized virgin PE microspheres [46]. Whether recycled LDPE toxicity potential differs from that of virgin LDPE, in particular in sensitive biological endpoints such as change of some immune parameters which has previously been shown to be changed by other types of MP, such as polyester fibres and tire wear particles [47] remains an open question.

The aim of this study was to provide long-term toxicity data of virgin *vs* recycled LDPE MP for two commonly used ecotoxicity models, the freshwater crustacean *Daphnia magna* and the terrestrial crustacean *Porcellio scaber*. In both organisms, the main exposure route is ingestion and both have a rigid exoskeleton, made of a multi-layered cuticle and covering body surfaces and ectodermal parts of the digestive system. The cuticle has different functions based on species-specific morphology, including primarily defense but also reception of external stimuli, such as chemicals (including MP). We compared whether recycled LDPE, which potentially has a more complex chemical composition than virgin LDPE, induces more (significant) adverse effects in the form of MP. Particular focus was on the sublethal reproduction-related (*D. magna*), feeding, as well as on selected immune-related parameters (*P. scaber*). The parameters were chosen as they cover a wide range of potential MP-target systems and may upon long-term MP-exposure reveal some effects not commonly detected in acute exposure. This is the first long-term comparative study on the effects of MP for freshwater and soil organisms, both crustaceans.

## 2. Materials and Methods

### 2.1. Microplastics

Both virgin and recycled microplastics (MP) were used as model MP. According to the providers’ data, both types were low density polyethylene (LDPE), that was also confirmed in the study by ATR-FTIR analysis. Virgin LDPE MP originated from Icopolymers (ICO Polymers, a division of A. Schulman, Allentown, PA, USA) and was used as provided. Recycled LDPE was obtained from an Estonian packaging company according to which it was the recyclate (mechanical recycling) of transparent LDPE film (mostly label-free packaging film) with a density of 0.930–0.945 g cm^−3^. Differently from the virgin LDPE, recycled LDPE was obtained in the form of granules/pellets (Figure S1) and was thus further milled to obtain fragments, comparable in size to those of virgin LDPE. Milling of recycled LDPE was performed in two stages. First, the pellets were ground into fragments by a SM100 mill (Retsch GmbH, Haan, Germany) and were further cryo-milled using a homogenizer (MillMix 20, Domel, Železniki, Slovenia) according to [48]. Both LDPE were tested and analysed as irregular fragments (microfragments) (Figure S2).

### 2.2. Physico-Chemical Characterization of Microplastics

#### 2.2.1. Fourier Transform Infrared Spectroscopy

Polymer samples were analysed using Fourier transform infrared spectroscopy (FTIR) with an attenuated total reflection (ATR) accessory. ATR-FTIR transmittance spectra were measured in the 400–4000 cm^−1^ range with 1 cm^−1^ resolution using a VERTEX 70 spectrometer (Bruker, Billerica, MA, USA) and the OPUS 7.0 software (Billerica, MA, USA).

#### 2.2.2. Particle Size Analysis

The number and volume particle size distributions of MP were measured using a S3500 Bluewave laser diffraction particle size analyser (Microtrac MRB, York, PA, USA). Analyses were done on powder.

#### 2.2.3. Scanning Electron Microscopy

Scanning Electron Microscopy (SEM) imaging of the MP was performed using a Nova NanoSEM 450 (FEI, Thermo Fisher Scientific, Newington, NH, USA) at 5 kV. For SEM analysis, particles were dispersed in water and transferred onto a silicon wafer. After drying, the samples were covered with a 2 nm thick Au layer (Emitech Sputter Coater, Quorum Technologies, Ringmer, UK) for improved conductivity.

#### 2.2.4. Total X-ray Diffraction Fluorescence Spectroscopy

Total X-ray diffraction fluorescence spectroscopy (TXRF) (PICOFOX S2, Bruker Nano GmbH, Berlin, Germany) was used to quantify the metals in both MP as well as in the medium of *D. magna* exposure. 10 mg of both virgin and recycled LDPE was weighed (AD-2 Autobalance, Perkin-Elmer, Waltham, MA, USA) into Eppendorf tubes. Fifty µL of ultraclean HNO_3_ (Merck, Darmstadt, Germany) and 100 µl of 1 mg Ga/L (internal standard) were added to each sample and left overnight. Then, 5 µl of each sample was pipetted on sample holders and the liquid was evaporated at 80 °C. By repeating this procedure 5 times, the samples were concentrated. Since elevated concentrations of some metals were detected in the recycled LDPE, *D. magna* exposure medium was also randomly sampled from two of the highest recycled LDPE treatments (100 mg/L) and the respective controls. 40 µl of sample was mixed with 40 µl of Ga standard (final concentration 1 mg Ga/L) after which the samples were concentrated 10 times as described above and analysed. Metal concentration in the sample was calculated using the Bruker Spectra7 software (Bruker Nano GmbH, Berlin, Germany).

#### 2.2.5. Gas Chromatography and Mass Spectrometry Analysis

Gas chromatography and mass spectrometry (GC-MS) analysis was used to identify organic compounds in the LDPE. For that, 0.2 g of LDPE was weighed into vial and 0.6 g of methanol was added, spiked with the exact amount of diethyl adipate as internal standard. The exact mass of spiked methanol was recorded. The vial was sealed, put in an autoclave and heated for 48 h at 100 °C. After the autoclave was cooled, vials were removed and centrifuged at 9000 rpm. A small amount of methanol solution was transferred into 0.2 mL GC-MS vial with a syringe and sealed. GC-MS analysis was performed on a 7890B gas chromatograph (Agilent, Santa Clara, CA, USA) coupled with a 5977B quadrupole mass detector. GC-MS analysis conditions were as follows: column: Agilent DB-5 MS Ultra Inert (Santa Clara, CA, USA), injected volume: 1 μL, inlet temperature: 250 °C, carrier gas: He, split ratio: 1:30. Temperature program: Initial temperature: 45 °C, hold time: 5 min, ramp rate: 10 °C/min, final temperature: 250 °C, hold time: 10 min. Components were identified based on mass spectra compared to National Institute of Standards and Technology (NIST) standards. Quantitative analysis was performed based on the peak area of each component compared to the peak area of the internal standard. Relative response factor of 1 was used in calculation.

### 2.3. Toxicity Testing

#### 2.3.1. Daphnia magna Chronic Reproduction Assay

OECD211 guidelines [49] were followed in the 21-day *D. magna* reproduction assay. Natural water from Lake Raku (Tallinn, Estonia) was used as the exposure medium. The water was collected in March 2020, filtered using a 0.45 µm cellulose nitrate filter (sterile) and stored at +4 °C. The analysis of selected physico-chemical parameters of the filtered water was performed in Tallinn Water Ltd. Laboratories (Tallinn, Estonia). Parameters of the natural water were the following: conductivity (20 °C) (µS/cm) 224; pH 8.41; dissolved organic carbon (mg C/L) 13.6; total hardness (mmol/L) 1.22; total phosphorus (mg P/L) 0.065; total nitrogen (mg N/L) 0.30; chloride Cl^−^ (mg/L) 4.1; ${\mathrm{SO}}_{4}^{2-}$ (mg/L) 14; S^2−^(mg/L) 0.006; Ca^2+^ (mg/L) 40.1; Mg^2+^ (mg/L) 4.55; Na^+^ (mg/L) 3.18; Ba^2+^ (μg/L) 134; K^+^ (mg/L) 1.56.

Both virgin and recycled LDPE MP were tested as concentrations of 1 mg/L, 10 mg/L and 100 mg/L. No surfactants were used to disperse the MP that was individually weighed (Perkin-Elmer AD-2 Autobalance, Waltham, MA, USA) into each test beaker with ≤8%, 6% and 5% of variability for 1 mg/L, 10 mg/L and 100 mg/L, respectively. Each LDPE was tested in 2 independent assays in 15 technical parallels/each. Mortality, reproduction, body length and total lipid content of daphnids were registered as toxicity endpoints. To measure the body length and monitor the daphnids’ morphology, a SMZ1270 stereomicroscope (digital camera DS-Fi3 colour camera 5.9 MP CMOS, software NIS-BR, Nikon Corporation, Tokyo, Japan) was used.

#### 2.3.2. *Daphnia magna* Lipid Quantification with Nile Red

Lipid quantification of adult daphnids from recycled-LDPE exposure was following the procedure from [50]. Nile Red (99% pure, ACROS Organics™, Geel, Belgium) (NR) stock solution was prepared in acetone (≥99.5%, Sigma-Aldrich, Steinheim, Germany) and stored in the dark at +4 °C. Prior to lipid staining, the NR stock was freshly diluted into OECD202 artificial freshwater (AFW) (mg/L of DI water: 294 CaCl_2_•2H_2_O, 123.25 MgSO_4_•7H_2_O, 64.75 NaHCO_3_, 5.75 KCl; pH 7.8 ± 0.2) at 1 mg/L and at the end of the chronic exposure, adult daphnids were individually stained for 1 h at room temperature in the dark. After 5 min rinsing in AFW, daphnids were individually transferred to 300 µl 2-propanol (≥99.5%, Merck, Darmstadt, Germany) and disintegrated by UH probe (Branson 450 Digital Sonifier Branson, Danbury, CT, USA) sonication (40 W, 10 sec). Upon centrifugation (10,000 g, +4 °C, 5 min), fluorescence of the 200 µl supernatants was measured (ex/em 530/590 nm) by Ascent Fluoroscan (Thermo Fisher Scientific, Vantaa, Finland). In total, 30 adult daphnids/sample were analysed and the obtained relative fluorescence values were compared with those of the untreated control sample. Lipid quantification from virgin LDPE MP-exposed daphnids was not performed due to technical reasons.

### 2.4. Terrestrial Isopods, Woodlice Porcellio scaber

#### 2.4.1. Test Organisms

Terrestrial isopods, woodlice *Porcellio scaber*, were collected from a compost heap in a non-contaminated, pollution-free garden in Kamnik, Slovenia (46°13′32.988′′ N; 14°36′42.12′′ E). Before the experiments, they were cultured for several months under constant temperature (20 ± 2 °C) and illumination (16:8 h, light:dark) in a climate-controlled chamber. They were caged in glass containers with a mixture of loamy sand and peat at the bottom (at 40% water holding capacity-WHC), and fed on dry leaves from common hazel (*Corylus avellana*) and common alder (*Alnus glutinosa*), and on carrots, as described by [40]. The soil and dry leaves were dry sterilised at 105 °C for 3 h before the woodlice were introduced into the glass containers. Only healthy, adult woodlice (30–60 mg fresh body mass) of both sexes were used. Molting woodlice, females with marsupia, and those showing symptoms of bacterial or viral infection, were excluded.

#### 2.4.2. Experimental Design

A 3-week soil exposure test was performed. For each of the experiments, the following concentrations of MP in standard agricultural soil (Lufa 2.2; Speyer, Germany, Table S1) were prepared: 0.02%, 0.06%, 0.17%, 0.5% and 1.5% *w*/*w* (0.2–15 g LDPE/kg soil). For a control group, soil without added MP was used. MP was first mixed with the dry soil and after a day of stabilization the moisture content was adjusted to 40% of the WHC by addition of deionised water and mixing. The soil was transferred into the test jars (15 g of moist soil in each 41 mL jar). Fifteen replicate jars were prepared for each treatment and control. In each test jar, one woodlouse was introduced and dry common hazel leaf was added for food. Altogether, 90 woodlice were used in this study for all treatments for each MP type, including the control (altogether 180). We noticed that the addition of MP in soil may affect the soil moisture as shown by our preliminary experiment where virgin LDPE increased the water loss during exposure, while recycled LDPE decreased the water loss in comparison to control (Figure S3). Therefore the soil moisture was checked daily and adjusted to desired 40% WHC.

#### 2.4.3. Survival, Feeding and Haemolymph Immune Parameters

Survival of animals was assessed daily, but shown as cumulative mortality after 3 weeks. Feeding activity of the animals was assessed after each week of exposure, when the leaves were replaced with new leaves. Feeding was calculated as a difference between initial and final dry leaf mass per animal per week (mg food/mg animal/week). The total number of samples analysed for feeding was 11–15 per treatment, depending on the number of surviving animals.

Selected immune parameters (total haemocyte count-THC, number of granulocytes and semigranulocytes, haemocyte viability) in woodlice haemolymph were checked only at the end of the 3-week exposure. Methods are described in detail in [51]. After the experiment, 5 µL of the haemolymph was collected from a single woodlouse or pooled from multiple woodlice using a sterile syringe and a glass micropipette (Brand, Wertheim, Germany). Freshly collected haemolymph was immediately diluted in Dulbecco Phosphate Buffer Saline (DPBS, pH 7.1–7.5) and used for measuring cellular immune parameters, e.g. total haemocyte count, differential haemocyte count and haemocyte viability using Neubauer haemocytometer (Brand, Wertheim, Germany) under light microscopy (Axio Imager Z1; Zeiss, Oberkochen, Germany), as described by [51]. Eight haemolymph samples per treatment were analysed for THC, granulocytes, semigranulocytes, and haemocyte viability.

#### 2.4.4. Statistical Analysis

Statistical differences between untreated *D. magna* control and LDPE-exposed samples were evaluated in one-way ANOVA. The data obtained for woodlice were visualized and analysed using OriginPro v2020 software (OriginLab, Northampton, MA, USA). For normal distributions and homoscedasticity of the data, one-way ANOVA was performed followed by Tukey tests; otherwise, a non-parametric Kruskal-Wallis test was used, followed by Mann-Whitney U-tests (Tables S2 and S3). *p* < 0.05 (*) was considered as significantly different.

## 3. Results

### 3.1. Characteristics of Microplastics

In the FTIR-ATR analysis, absorption bands identified in the FTIR spectra of both polymeric samples were all characteristic of low-density polyethylene (LDPE) [52] (Figure S4). LDPE was also confirmed by the built-in spectral library of the OPUS software. In the ATR-FTIR spectrogram (Figure S4), 2400 cm^−1^ and 670 cm^−1^ peaks in the recycled LDPE spectra originate from CO_2_ (atmospheric CO_2_ from the measuring chamber but potentially also from CO_2_ adsorbed on the polymer surface).

**Figure 1:**
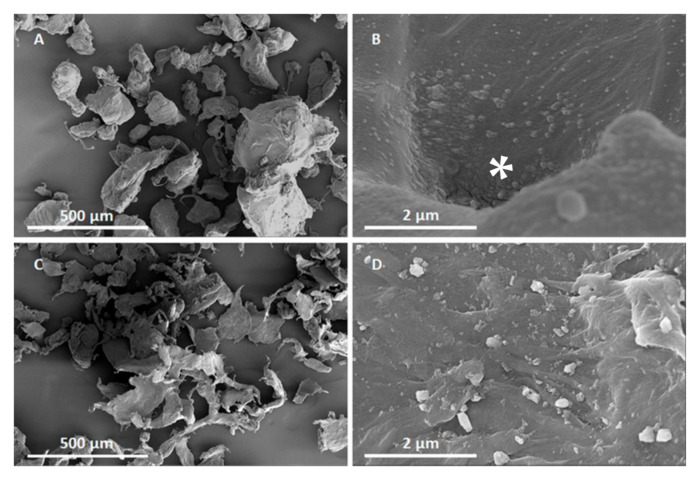
Scanning Electron Microscope (SEM) images of virgin (**A**,**B**) and recycled LDPE (**C**,**D**) microplastics. SEM images reveal highly variable particle shapes. Higher resolution images of particle surfaces (**B**,**D**) indicate a possible <100 nm sized particle fraction, that in virgin LDPE are mainly associated with pits (indicated with *). Scale bar equals 500 µm (**A**,**C**) and 2 µm (**B**,**D**).

Scanning Electron Microscopy (SEM) imaging revealed particles were highly irregular with wide size and shape variability (Figure 1). According to the SEM analysis, the majority of LDPE MP were in the ~30–300 µm size range, but particle-like material as small as 30 nm could be seen on the particle surfaces for both virgin and recycled LDPE (Figure 1). In recycled LDPE, the nanosized structures on the surface of bigger particles occurred rather regularly (Figure S5). In virgin LDPE, these structures were mostly observed associated within certain irregularities as the pits, featured in Figure 1. Whether and to what extent the nanosized structures were detachable from the surface of larger particles was not determined.

**Figure 2:**
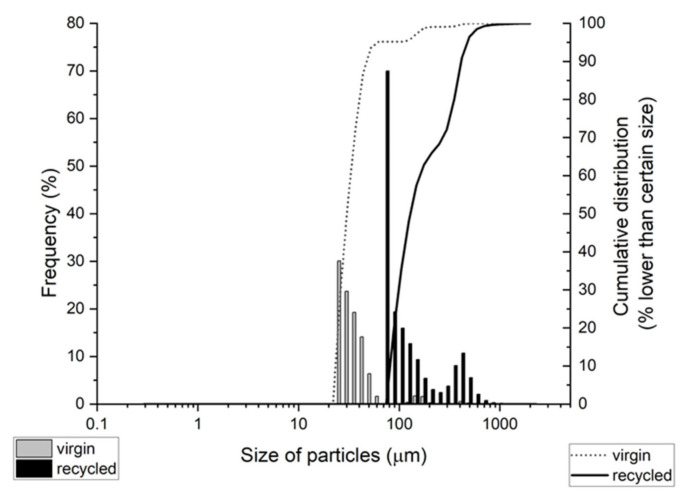
Particle size distribution according to laser diffraction particle size analysis. The share of particles of certain size (frequency, columns; left y-axis) and cumulative distribution (line, right y-axis) are presented.

The mean size range of particles from laser diffraction particle size analysis was 39.8 ± 8.8 µm for virgin LDPE and 205 ± 144 µm for recycled LDPE (±SD; number distribution). Cumulative distribution showed that 95% of virgin LDPE MP was in the range of 26–125 µm and 95% of recycled LDPE MP was in the range of 88–418 µm (Figure 2). The lowest particle detection size for this analysis was 290 nm, hence no nanoparticles that were observed in SEM could be detected by laser diffraction analysis.

Metal analysis of the two LDPE samples by TXRF showed that the concentration of Ca and Fe were 59 and 400-fold higher, respectively in the recycled LDPE compared to the virgin LDPE (Table 1). In addition, the concentrations of toxic metals Cu and Pb were 12-fold and 55-fold higher, respectively in recycled LDPE. Despite these elevated concentrations in the recycled LDPE, no metal leaching was detected at the highest exposure concentrations (100 mg LDPE/L) at *D. magna* assay conditions (Table S4). Potential contamination of the recycled LDPE with Ca and Fe in the milling process cannot be ruled out however, the recycled LDPE pellets were yellow-brown (Figure S1) which is also potentially an indication of high Fe content.

GC-MS analysis identified (with over 65% quality match) six different methanol-extractable organic compounds in virgin and recycled LDPE yet the concentrations were significantly higher in virgin LDPE (Table 2). The highest concentration in virgin LDPE was observed for benzenepropanoic acid, 3,5-bis(1,1-dimethylethyl)-4-hydroxy-, methyl ester (5.33 mg/g LDPE) and in recycled LDPE for hexanedioic acid, ethyl methyl ester (0.09 mg/g LDPE). Chromatograms of the GC-MS results are included in the SI (Figure S6).

### 3.2. Daphnia magna Reproduction

*D. magna* reproduction assays showed that neither virgin nor recycled LDPE MP induced any parental mortality, significant (*p* > 0.05) reproductive impacts, parental organism body length nor total lipid content differences compared to the respective unexposed control organisms (Table 3). Coefficient of variation (CV) % of the mean number of living offspring/parent daphnid throughout the assays was 10–17% thus in line with the OECD211 test guideline requirements (≤25%).

The only adverse effects for MP-exposed *D. magna* were recorded in one of the recycled LDPE exposure where 1.3% (5/385 neonates) and 1.1% (4/374 neonates) neonatal mortality was observed for 10 mg/L and 100 mg/L exposures to recycled LDPE, respectively. In addition, in the 100 mg/L sample, an embryo, retained in the shed carapace, (Figure S7) was recorded. No visible LDPE particles were noticed on the carapace, containing the embryo. Some potential morphological abnormalities of the embryo (Figure S7) were observed. Another dead neonate was recorded in the same sample yet the cumulative reproduction per this parental organism was 104% of the unexposed control average reproduction/daphnid. In control and virgin LDPE-exposed samples, no dead offspring were observed.

### 3.3. Woodlice Porcellio scaber Responses

#### 3.3.1. Feeding and Mortality

Neither MP (virgin and recycled) significantly affected the survival of woodlice. The highest mortality was 20% which is still within the range allowed for controls in experiments with isopods [53]. No difference in mortality was observed between groups exposed to virgin or recycled LDPE.

**Figure 3:**
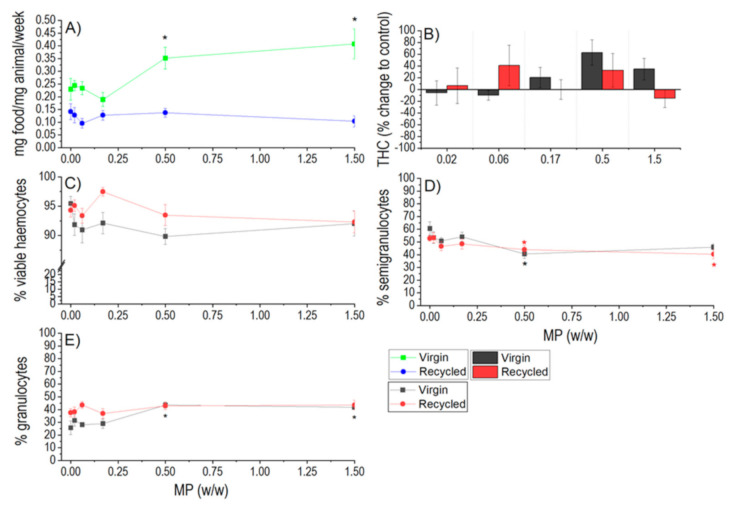
Feeding rate (**A**) and immune response (**B**–**E**) of woodlice after 3 weeks of exposure to virgin and recycled LDPE microplastics. For immune response, total haemocyte count (THC), proportions of different types of haemocytes (granulocytes, semigranulocytes), and viability of haemocytes are shown. Average values (AVG ± SE) are shown. Asterisks indicate statistically significant differences in comparison to respective controls (*) (*p* < 0.05).

A significant increase in feeding activity on non-contaminated leaves was observed for virgin LDPE at the two highest concentrations (0.5% and 1.5% *w*/*w*), but the feeding was not affected after exposure to recycled LDPE. The two control groups, virgin and recycled LDPE, exhibited slightly different feeding rates but the difference was not statistically significant (Figure 3A).

#### 3.3.2. Immune Parameters

In the case of virgin LDPE the total haemocyte count was not statistically significantly different from controls due to high variability of data, but a trend of increase by 63% and 35% in comparison to control was observed at 0.5% and 1.5% *w*/*w*, respectively (Figure 3B). A significant increase in granulocytes and decrease in semigranulocytes were found at the two highest concentrations of virgin LDPE (0.5% and 1.5% *w*/*w*). Viability of haemocytes was not affected in this treatment. In the case of recycled LDPE, similarly no significant changes in total haemocyte count were found, but 42% and 31% increase in comparison to control was observed at 0.06% and 0.5% *w*/*w*. The haemocyte viability and share of granulocytes were not changed (Figure 3C,E), but the share of semigranulocytes was decreased at the two highest concentrations (0.5% and 1.5% *w*/*w*) (Figure 3D). This means that both types of LDPE at high exposure concentrations induced a slight shift in selected immune parameters evidenced by the total haemocyte count and in the change in their proportions with more parameters altered in the case of the virgin LDPE.

## 4. Discussion

In this study we report biological response (toxicity) data for realistic microplastics (MP) types in ecologically relevant long-term exposures that could be of high applicability for risk assessment purposes [54] As the MP, LDPE fragments, the most often observed MP types in freshwater [55,56], were chosen. While fibres represent the major portion of MP sampled in agricultural soils amended with sewage sludge or biosolids [26,57], films and fragments have also been frequently recovered [57].

A wide span of LDPE concentrations (1–100 mg/L for *D. magna* and 200–15,000 mg/kg for *P. scaber*) was chosen to characterize both the current as well as predict the impact for future scenarios in the business-as-usual plastic usage conditions. Since soils have been shown to be sinks of MP and the concentrations are predicted to increase in the future, higher exposure-scenario concentrations were chosen for *P. scaber* assays. Environmental MP concentrations have been shown to vary greatly, reflecting the potential influence of site- as well as monitoring-specific factors. For instance, in soil, 7100–42,960 [57] and 320–12,560 [58] MP/kg in agricultural and 0.3–67 g MP/kg; corresponding to 0.03–6.7% *w*/*w*) in industrial areas have been reported [59]. Likewise, for freshwater, within a single monitoring study, highly variable MP abundance of 43,157 ± 115,519 MP/km^2^ (0–466,305 MP/km^2^) [34] in the Laurentian Great Lakes (US) was recorded. In the marine environment, LDPE has been shown to dominate the sea surface (42%) but to be very sparse (2%) in the deep-sea [60]. In the current study, no chemicals were used to disperse the buoyant LDPE fragments in *D. magna* assays and since the media was changed every third day, the fragments were afloat in test vessels thus mimicking LDPE MP contamination in nature and limiting MP ingestion by daphnids in the test settings.

There were no differences (*p* > 0.05) in parental *D. magna* body length, reproduction or total lipid content (only performed for recycled LDPE) from the untreated organisms. No external damage for *D. magna* was recorded for both LDPE and very small inconsistent effects (1% neonatal mortality and one unborn embryo, retained in shed carapace) were recorded in a recycled-LDPE experiment (at 10 and 100 mg MP/L). Likewise, in the exposure to irregular ~40 µm MP (mix of raw polymers of minimal chemical composition), no adverse effects (survival, reproduction, morphology) for parental and subtle morphological adverse effects for juvenile daphnids were detected [43]. In contrast, irregular MP (10–75 µm) of recycled black PE were found to induce immobilization and complicated egestion in *D. magna* but the effects were rather attributed to the particle shape as potential influence of additives was not studied [46]. Most of the MP hazard data however originates from studies where spherical MP have been used and a variety of adverse effects from long-term exposures has been shown. For *D. magna*, mortality [61], decreased reproduction [61,62,63,64] decreased growth and population growth rate [62] has been shown for virgin 1–6 µm MP spheres (polymeric composition not reported, PE and PS) from concentrations as low as 0.1 mg/L [62]. Similarly, adverse effects on *Daphnia* longevity, growth, reproduction [65,66] and physiological endpoints (heart and appendage beat rate) [44] have also been demonstrated upon PS nanosphere exposures. Physical properties of the MP are essential regarding inducing of adverse effects [46] and facilitated MP uptake by various routes (orally, anally) by *D. magna* (or other organisms) and moreso, inhibited egestion may be one of the most important triggers leading to adverse effects, especially over a longer exposure period. Decreased reproduction has indeed been hypothesized to be a consequence of impaired feeding and nutrient assimilation upon PS MP internalization [64,66] and has been demonstrated by 3-fold higher intake and more significant adverse effects of palmitic acid-functionalised PS spheres compared to non-functionalised ones [66]. In our study, virgin LDPE fragments (mean size 39.8 ± 8.8 µm) were theoretically ingestible by *D. magna* [67] whereas 95% of the recycled LDPE fragments in the range of 88–418 µm were not. However, also in the case of recycled LDPE ingestion of MP cannot be ruled out in particular if a fragment would be narrow enough. For example, even a 1400 µm long polyester fiber (approximate width 40 µm) was found in the gut of *D. magna* [68]. According to SEM imaging, nanosized fraction was observed in recycled LDPE but it was not determined whether it was bound to larger fragments and was thereby bioavailable to *D. magna*. However foremost due to the buoyancy and irregular shape of the LDPE MP, the uptake by daphnids was not expected to be systematic nor was MP adsorbance on daphnids seen in light microscopy observations.

As in the current study, immobility of *Daphnia* offspring from chronic exposure but a significantly higher one was recorded upon exposure to 5 µm MP spheres (up to 52% immobility) as well as to 5 nm gold nanoparticles (up to 75% immobility) [61] thus the effect was not limited to a material nor particle size. The fact that in the current study, nanosized fraction was noted on recycled LDPE (Figure S5) raises the question on the impact of particle size and availability for organismal uptake and on the concurrent effects. For instance, association with various parts of *D. magna* carapace has again been demonstrated only with spherical MP [66]. Despite the different potential to affect apical toxicity endpoints, altered gene expression of *Daphnia* (mostly stress-related genes) has been shown for both irregular [43] and spherical MP [65] exposures. Regardless of the MP shape, all the discussed studies except [46] have been conducted on virgin polymers.

For *P. scaber*, a significant increase in feeding activity was observed for virgin LDPE (39.8 ± 8.8 µm) at the two highest concentrations (0.5% and 1.5% *w*/*w*) (5 and 15 g MP/kg soil), but feeding was not affected after exposure to recycled LDPE (205 ± 144 µm). Woodlice do not have a certain particle-size ingestion limitation as they are shredders of litter in contrast to daphnids which are filter feeders. They could in fact fragment macroplastic to microplastic, which has not been yet shown for woodlice, but for other crustaceans [69] and earthworms [70]. Usually, a decrease of feeding upon exposure to MP is anticipated due to clogging of the digestive tract and damage of intestinal epithelium [39]. In parallel, decreased growth and depletion of energy reserves are commonly observed [35,71]. A similar finding, that is decrease of feeding and energy reserves were observed at 0.06%, 0.5% and 1.5% *w*/*w* of polyester fibres [48]. On the contrary, LDPE fragments exposure in this study increased the feeding of woodlice. Increased feeding of invertebrates has previously been observed upon exposure to metals [72] and organic chemicals [73]. A plausible explanation is that increased feeding rate is a feedback loop to increased metabolic rate required to directly combat deleterious effects of contaminants, as seen from experiments on crayfish [74]. What exactly caused this effect in woodlice, either the particles, their associated chemicals or a mixture of both is difficult to establish. In any case, it has to be mentioned that woodlice increased the feeding of uncontaminated leaves that were placed onto MP-contaminated soil, meaning that the exposure in soil induced some physiological changes that led them to consume more food and it was not the food quality that might affect their feeding. The highest concentrations of organic chemicals in virgin LDPE were found for 3,5-bis(1,1-dimethylethyl)-4- hydroxybenzenepropanoic acid methyl ester and methyl ethyl adipate, however their final nominal concentrations in soil anticipating their maximum release from particles to soil would be negligible (0.008% and 0.003% *w*/*w* soil, respectively).

In woodlice, we also observed a change of selected immune parameters upon exposure to both virgin and recycled LDPE. This was evidenced in particular by an increase in granulocyte count and decrease in semigranulocytes at the two highest concentrations (0.5% and 1.5%) of virgin LDPE and decrease of semigranulocytes at 1.5% *w*/*w* recycled LDPE. Such opposite trends in granulocyte and semigranulocyte counts are frequently observed, as granulocytes and semigranulocytes appear to represent two consecutive phases of haemocyte maturation [75]. A shift in metabolic processes may lead to the formation of granules, and thus to an increase in the granulocytes [47]. In both treatments a sporadic dose-independent trend in increase of the total haemocyte count was observed. This parameter is commonly increased upon microbial infection [51] or chemical (incl. MP) [47], albeit it is quite variable in a given population and hence difficult to prove differences among treatments statistically. Altogether all changes in immune parameters observed in this study indicate that immune response was induced upon exposure to both LDPE MP. The most plausible mechanism behind this is the alterations to the microenvironment of the gut; e.g., due to changes in gut chemistry and microbiome [36], responses to the changed diet [76], or an immune response to damage of the gut cuticle [77]. In comparison to the same concentrations of polyester fibres or tire wear particles that were exposed to *P. scaber* previously [47], the induction caused by virgin LDPE was more pronounced. Again, virgin LDPE seems to induce more changes than recycled LDPE, analogously to the higher effect on the feeding rate.

Unlike polymers such as polyvinyl chloride [21,78], LDPE is generally less expected to cause chemical toxicity. Although in the current study virgin LDPE was anticipated to be of simpler chemical composition than recycled LDPE, it was not. Instead, GC-MS analysis showed the identified organic compounds at much higher concentrations in virgin than in the recycled LDPE (Table 2). It is possible that in the process of recycling, organic compounds are (partially and/or selectively) removed in the cleaning and/or extrusion steps of the recyclate but GC-MS sample extraction methods may also impact the results. In Horodytska et al. [19], using combined headspace and solvent extraction, 79 volatile and semi-volatile organic compounds (match quality >65%) were GC-MS identified in recycled pellets, produced from post-consumer-LDPE waste (LDPE bags and films). In the current study, six (four of which were >80%) and six (one of which was >80%) organic compounds provided a reliable match (>65%) for virgin and recycled LDPE, respectively. Since in the current study, only solvent extraction was used for the GC-MS analysis, the results likely underestimate the whole range of organic compounds in the LDPE. There were no overlapping compounds (of over 65% quality match) in virgin and recycled LDPE in the current study nor were there any common compounds to those reported in the post-consumer LDPE by Horodytska et al. [19]. Contrary to organic compounds, metal concentration was higher (e.g. 12-fold for Cu, 55-fold for Pb, 59-fold for Ca and 400-fold for Fe) in recycled than in virgin LDPE (Table 1). The possible contamination of the recycled LDPE from the milling of pellets for toxicity analysis cannot be ruled out however, the recycled LDPE pellets were yellow-brown (Figure S1) that could also indicate high internal Fe content. The high Fe content in recycled LDPE could also have originated from mechanical recycling process (e.g. shredding, introduction of fillers) yet in Eriksen et al. [79] the differences in metal content were rather associated with material chemistry than physical contamination. Pb in recycled LDPE may have originated from pigments [80] although according to the manufacturer, the recycled packaging material was mostly label-free. Due to the increasing plastic recycling rate, future hazard evaluation of recycled plastics should also address its metal content since elevated concentrations, especially those of highly toxic metals (e.g. As, Cd, Hg, Pb), could pose a risk to the health and the environment [81,82].

## 5. Conclusions

This is the first study to compare the potential hazards of recycled and virgin LDPE microplastics (MP) using the freshwater and terrestrial crustaceans *Daphnia magna* and *Porcellio scaber*. Long-term exposure to irregular LDPE MP of 39.8 ± 8.8 µm (virgin) and 205 ± 144 µm (recycled) induced only minor effects in *D. m*agna and *P. scaber*. For *D. magna*, parental survival, reproduction, body length and organismal lipid content were comparable to the untreated control organisms at exposures of 1–100 mg LDPE/L. The causes for the small inconsistent mortality of *D. magna* offspring that occurred at higher concentrations of recycled LDPE are still to be determined. For *P. scaber*, virgin LDPE caused a remarkable increase in feeding and shift in the measured immune parameters at two highest concentrations (0.5% and 1.5% *w*/*w*), while recycled LDPE provoked only a slight change in immune parameters. This means that the 3-week exposure to both LDPE MP provoked some physiological responses in woodlice for which the significance with respect to fitness of the organism in the long-term is yet to be determined. The results of the study indicated different sublethal responses upon exposure to recycled compared to virgin LDPE MP. Contrary to our expectations, the chemical composition of recycled LDPE was not more complex than that of virgin LDPE and the identified (in)organic additives in both LDPE were not anticipated to induce the observed biological responses. From the obtained data, recycled LDPE MP (from mechanical recycling) was not more hazardous for *D. magna* and *P. scaber* than virgin LDPE MP. We suggest that in order to build trust in circular economy, comparative hazard evaluation of polymers, originating from different recycling methods, and their virgin analogues should be systematic.

## Figures and Tables

**Table 1:** Concentrations of selected metals (mg metal/kg) in LDPE (virgin and recycled) microplastics. LOQ–Limit of Quantification. LOQ (Pb)= 0.01 mg/L. Data are given as AVG ± SD, *n* = 2.

	LDPE	
Metal	Virgin	Recycled
P	1.28 ± 0.69	5.18 ± 1.26
K	6.89 ± 9.13	22.9 ± 11.2
Ca	5.85 ± 4.58	347 ± 2.86
Ti	0.11 ± 0.07	0.21 ± 0.29
Fe	0.45 ± 0.22	181 ± 16.8
Cu	0.73 ± 0.71	8.87 ± 5.95
Zn	3.30 ± 1.61	3.89 ± 0.57
Pb	<LOQ	0.55 ± 0.42

**Table 2:** Identified (only matches >65% have been included) and quantified (mg/g) organic compounds from LDPE (virgin and recycled) methanol-extractions.

	Organic Compound	RT	% Match	mg/g LDPE
**Virgin** **LDPE**	Benzenepropanoic acid, 3,5-bis(1,1-dimethylethyl)-4-hydroxy-, methyl ester	22.561	97.2	5.33
	Methyl ethyl adipate	15.225	95.9	2.09
	Butanedioic acid, dimethyl ester	10.618	95.8	1.52
	Methyl adipate	14.182	95.7	1.29
	Butylated hydroxytoluene	17.833	66.3	1.06
	Hexadecanoic acid, methyl ester	22.5	72.8	0.33
	Diethyl adipate*	16.203	80.8	0.53
**Recycled LDPE**	Hexanedioic acid, ethyl methyl ester	15.225	94.8	0.09
	Oleamide	26.577	79.2	0.04
	Dimethyl terephthalate	17.755	67.0	0.03
	2,6-Bis(1,1-dimethylethyl)-4-methyl-4-isopropylcyclohexa-2,5-dien-1-one	17.833	68.2	0.01
	Benzoic acid, 3,5-bis(1,1-dimethylethyl)-4-hydroxy-	19.828	71.9	0.01
	3,3-Dimethyl-4-methylamino-butan-2-one	18.976	68.2	0.01
	Diethyl adipate *	16.203	88.4	0.56

*-Diethyl adipate was the internal standard; RT-retention time.

**Table 3:** Toxicity results of virgin and recycled LDPE plastic in *Daphnia magna* chronic reproduction assay. All data are presented as AVG ± SD (*n* = 2; 15 parallels). LDPE–low density polyethylene; RFU–relative fluorescence units (measured at 530/590 nm); n.d.–not determined.

	LDPE	Time to 1st Brood	Offspring/Female	Broods/Female	Total OFFSPRING/Treatment	Body Length	Total Lipids
	mg/L	(Days)	(Number)	(Number)	(Number)	(mm)	(RFU)
	0	10.5 ± 0.71	33.7 ± 13.3	3.50 ± 0.71	505 ± 199	3.30 ± 0.24	n.d.
virgin LDPE	1	10.0 ± 0.0	33.4 ± 13.7	3.50 ± 0.52	497 ± 211	3.25 ± 0.37	n.d.
	10	10.0 ± 0.0	33.1 ± 13.3	3.44 ± 0.33	497 ± 199	3.34 ± 0.21	n.d.
	100	10.0 ± 0.0	33.5 ± 12.8	3.45 ± 0.78	503 ± 192	3.32 ± 0.21	n.d.
	0	10.5 ± 0.71	27.4 ± 0.64	3.87 ± 0.09	410 ± 10	3.41 ± 0.09	2703 ± 1186
recycled LDPE	1	10.5 ± 0.71	27.2 ± 0.99	3.94 ± 0.09	409 ± 15	3.44 ± 0.08	2418 ± 413
	10	10.0 ± 0.0	26.8 ± 1.65	3.87 ± 0.09	403 ± 25	3.41 ± 0.07	2843 ± 1841
	100	10.5 ± 0.71	27.2 ± 1.11	4.0 ± 0.0	392 ± 30	3.40 ± 0.10	2725 ± 1189

## Supplementary Materials

The following are available online at https://www.mdpi.com/2073-4360/13/5/771/s1, Figure S1: Pellets (left) of recycled LDPE (recyclate of transparent mostly label-free packaging film) and the fragments (right) from the first milling (Retsch SM100). For the current study, fragments from the first milling were further milled into smaller (<500 µm) fractions (Figure S2), Figure S2: Virgin (A, C) and recycled (B, D) LDPE microplastics in stereomicroscope Nikon SMZ1270. Scale bar equals 0.5 mm, Figure S3: Change in soil moisture in the control and at different concentrations of microplastics exposure (virgin and recycled LDPE) after 4 days, Figure S4: ATR-FTIR spectra of virgin LDPE (yellow line) and recycled LDPE (black line) powders. Characteristic absorption bands (cm-1) and the corresponding vibration modes used to identify the polymers as LDPE are marked, Figure S5: Scanning Electron Microscope images of the surface structures of virgin (A, C) and recycled (B; D) LDPE microplastics. Higher magnifications (C and D) are taken from the insets (black squares) on the upper panel, Figure S7: An embryo, retained in the shed carapace of Daphnia magna that had been exposed (18 days until this observation) to 100 mg/L of recycled LDPE. Yellow arrow indicates potential abnormalities of the embryo. Scale bar equals 0.5 mm, Table S1: Properties of Lufa 2.2 test soil (Lufa, Speyer, Germany), provided by the supplier, Figure S6: GC-MS chromatogram of virgin (top) and recycled (bottom) LDPE. Methanol extraction was used to prepare the samples, Table S2: Collected test statistics on the effects of the virgin LDPE alone on the woodlice (Porcellio scaber) exposed to 0.02%, 0.06%, 0.17%, 0.5% and 1.5% (*w*/*w*) virgin LDPE in Lufa 2.2 soil. Normality was tested with Kolmogorov-Smirnov tests, and homogeneity of the variances was verified with Levene tests. For data with parametric distribution and homoscedasticity, one-way ANOVA followed by Tukey tests was performed, while for data with non-parametric distribution or not achieving homoscedasticity, non-parametric Kruskal-Wallis tests followed by pairwise comparisons with the control was performed using Mann-Whitney U-tests, Table S3: Collected test statistics on the effects of the recycled LDPE alone on the woodlice (Porcellio scaber) exposed to 0.02%, 0.06%, 0.17%, 0.5% and 1.5% (*w*/*w*) recycled LDPE in Lufa 2.2 soil. Normality was tested with Kolmogorov-Smirnov tests, and homogeneity of the variances was verified with Levene tests. For data with parametric distribution and homoscedasticity, one-way ANOVA followed by Tukey tests was performed, while for data with non-parametric distribution or not achieving homoscedasticity, non-parametric Kruskal-Wallis tests followed by pairwise comparisons with the control was performed using Mann-Whitney U-tests, Table S4: TXRF-quantified concentrations of selected metals in Daphnia magna chronic reproduction assay treatments (100 mg recycled LDPE/L and untreated D. magna control). Metal concentrations are given as mg metal/L (AVG ± SD, n = 2). LOQ–Limit of Quantification.
