# Supplementary material for: Long Term Exposure to Virgin and Recycled LDPE Microplastics Induced Minor Effects in the Freshwater and Terrestrial Crustaceans Daphnia magna and Porcellio scaber
Source: Polymers (Basel). 2021 Mar 2;13(5):771. doi: 10.3390/polym13050771 (PMC7958955; doi:10.3390/polym13050771)
Supplement: Supplementary file 1 [file polymers-13-00771-s001.pdf]

Supplementary information

Long Term Exposure to Virgin and Recycled LDPE Microplastics Induced Minor Effects in the Freshwater and Terrestrial Crustaceans *Daphnia magna* and *Porcellio scaber*

Anita Jemec Kokalj [1], Andraž Dolar [1], Jelizaveta Titova [2], Meeri Visnapuu [3], Luka Škrlep [4], Damjana Drobne [1], Heiki Vija [2], Vambola Kisand [3] and Margit Heinlaan [2,*]

[1] University of Ljubljana; Biotechnical faculty; Department of Biology; Večna pot 111; 1000 Ljubljana; Slovenia; anita.jemec@bf.uni-lj.si (A.J.K.); Andraz.Dolar@bf.uni-lj.si (A.D.); damjana.drobne@bf.uni-lj.si (D.D.)
[2] Laboratory of Environmental Toxicology; National Institute of Chemical Physics and Biophysics; Akadeemia tee 23; 12618 Tallinn; Estonia; jelizaveta.titova@kbfi.ee (J.T.); heiki.vija@kbfi.ee (H.V.); margit.heinlaan@kbfi.ee (M.H.)
[3] Institute of Physics; University of Tartu; W. Ostwaldi Str 1; 50411 Tartu; Estonia; meeri.visnapuu@ut.ee (M.V.); vambola.kisand@ut.ee (V.K.)
[4] Slovenian National Building and Civil Engineering Institute; Dimičeva ulica 12; 1000 Ljubljana; Slovenia
* Correspondence: margit.heinlaan@kbfi.ee

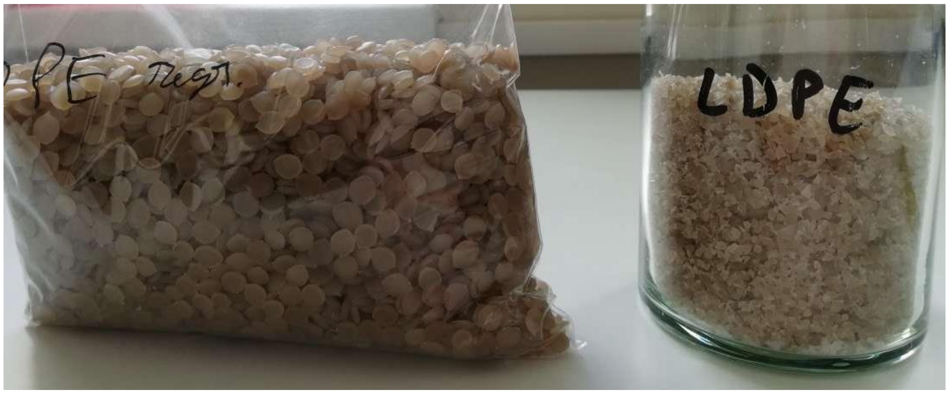

Figure S1. Pellets (left) of recycled LDPE (recyclate of transparent mostly label-free packaging film) and the fragments (right) from the first milling (Retsch SM100). For the current study, fragments from the first milling were further milled into smaller (<500 μm) fractions (Figure S2).

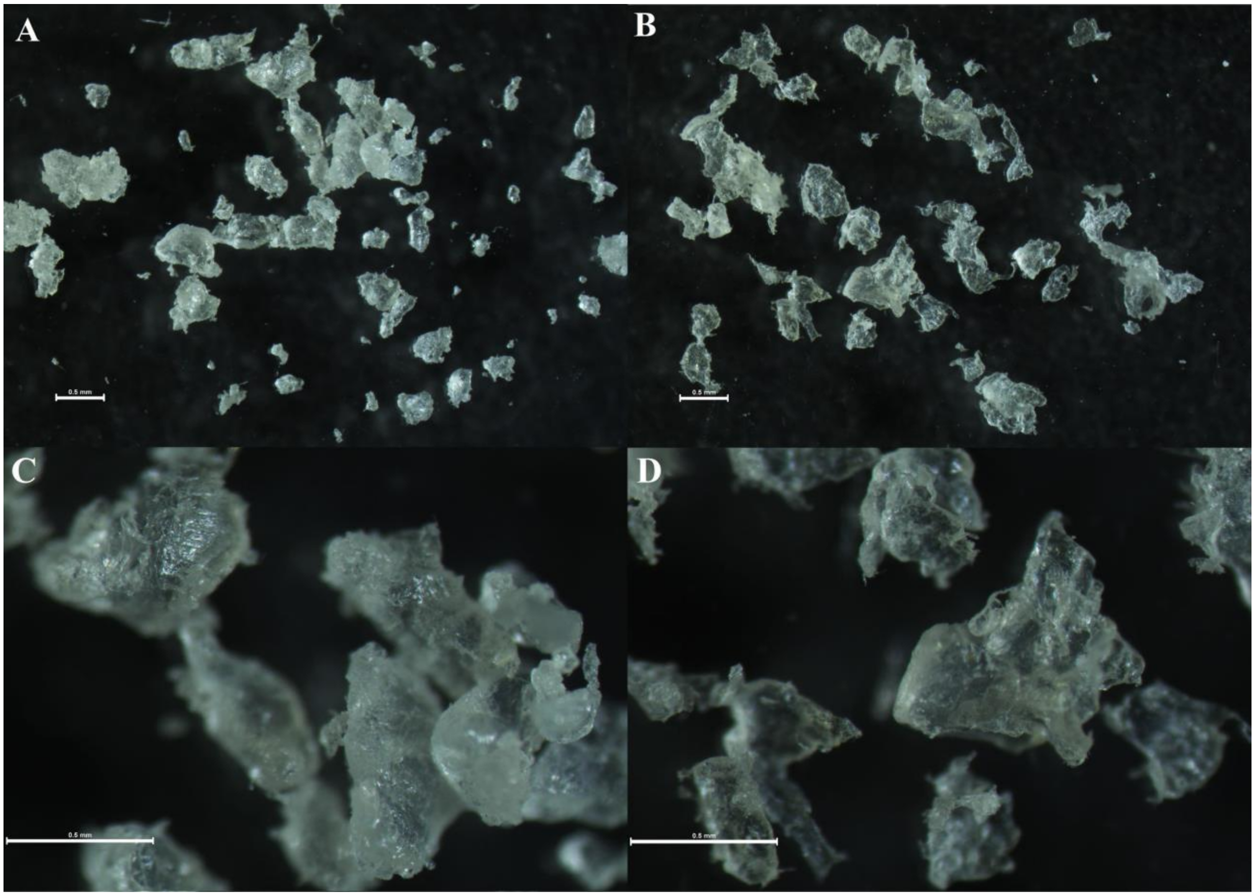

Figure S2. Virgin (A, C) and recycled (B, D) LDPE microplastics in stereomicroscope Nikon SMZ1270. Scale bar equals 0.5 mm.

Table S1. Properties of Lufa 2.2 test soil (Lufa, Speyer, **city, country**), provided by the supplier.

**Parameter**	**Mean ± Standard deviation**
Soil type	Loamy sand (DIN) / Sandy loam (USDA)
Organic carbon (% C)	1.73 ± 0.27
Nitrogen (% N)	0.19 ± 0.03
pH (0.01 M $CaCl_2$)	5.6 ± 0.4
Cation exchange capacity (cmolc kg^{-1})	9.8 ± 0.5
Water holding capacity (%)	45.8 ± 1.9
Weight per volume (g / 100 ml)	1201 ± 41

Effects of microplastics on soil moisture

In a preliminary experiment we noticed that the moisture in test jars with woodlice changed in a different manner than in the control exposure. Therefore we performed a separate preliminary experiment to check how the soil moisture changed if it was not adjusted to 40% water holding capacity. For this purpose, 3 jars per three selected microplastics (MP) concentrations (0.06%, 0.5% and 1.5% w/w) were filled with the same mass of soil as for the tests with animals. The jars were incubated for 4 days at constant temperature (20 ± 2 °C) and illumination (16:8 h, light:dark) in a climate-controlled chamber. We noticed that the loss of water was lower at 0.5% of recycled LDPE and higher at 1.5% w/w of virgin LDPE (Figure S3) although the difference could not be statistically tested due to low number of replicates (n=3). This indicates a possibility that virgin and recycled MP affect the soil property, which has also been previously shown for other types of MP [81]. In soil, LDPE fragments have been shown to harbor unique microbial communities that could potentially alter the soil ecological functions and biogeochemical processes [82]. The outcome of this preliminary experiment was taken into consideration during the exposure with woodlice. Special care was taken to adjust the replenished water to desired 40% water holding capacity on a daily basis.

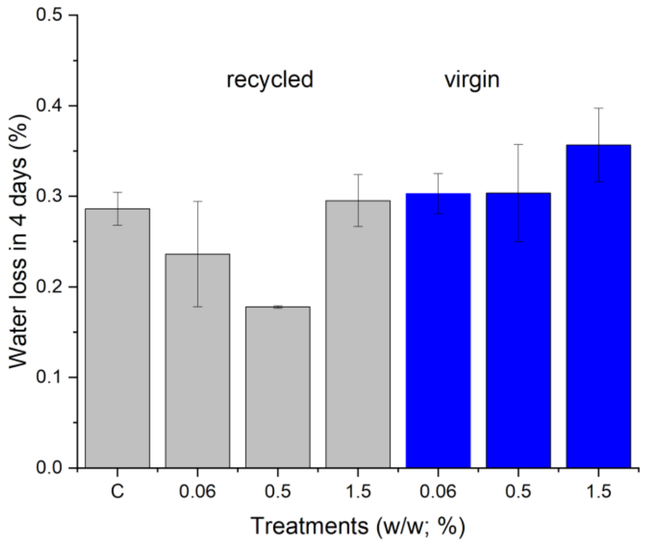

Figure S3. Change in soil moisture in the control and at different concentrations of microplastics exposure (virgin and recycled LDPE) after 4 days.

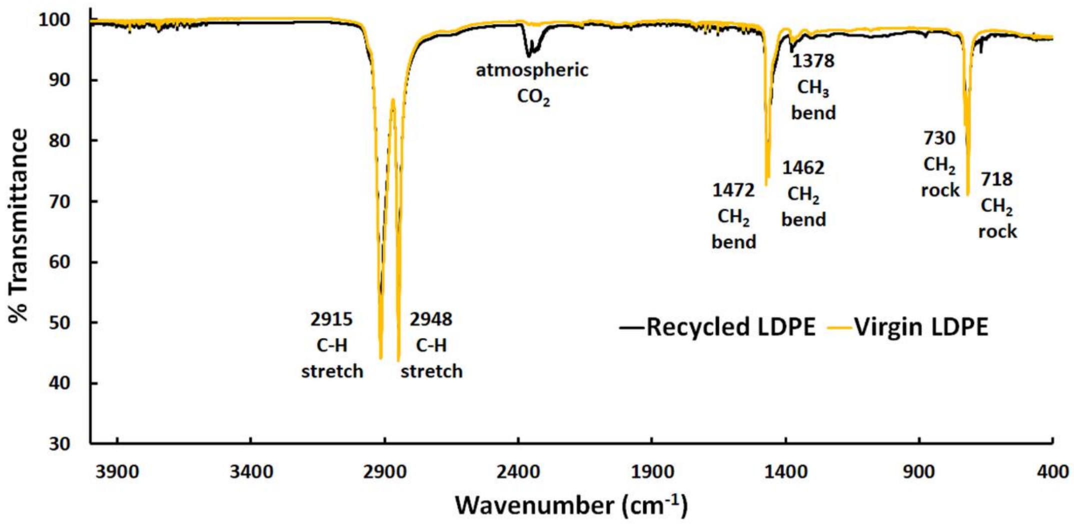

Figure S4. ATR-FTIR spectra of virgin LDPE (yellow line) and recycled LDPE (black line) powders. Characteristic absorption bands (cm^{-1}) and the corresponding vibration modes used to identify the polymers as LDPE are marked.

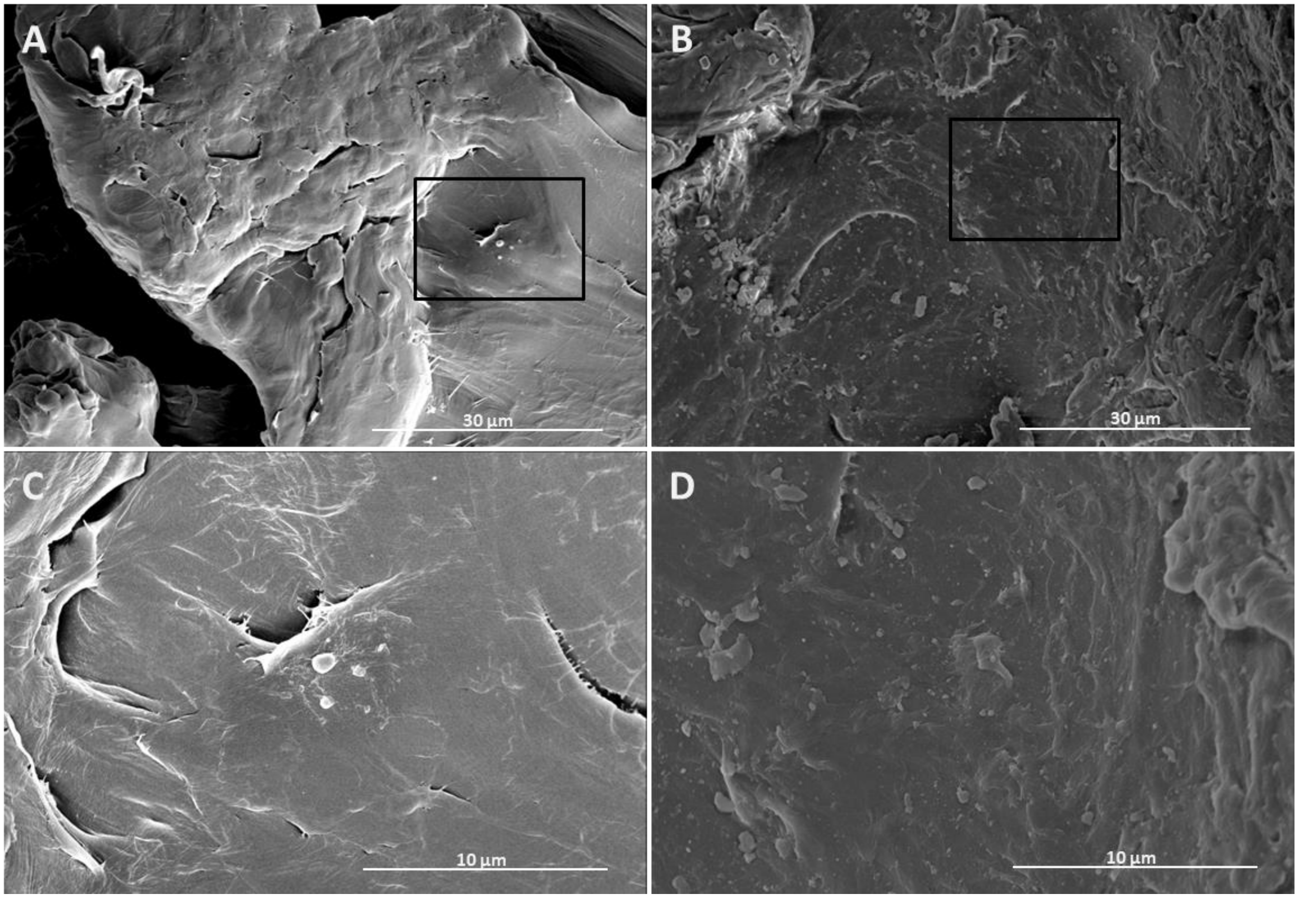

Figure S5. Scanning Electron Microscope images of the surface structures of virgin (A, C) and recycled (B; D) LDPE microplastics. Higher magnifications (C and D) are taken from the insets (black squares) on the upper panel.

Table S2. Collected test statistics on the effects of the virgin LDPE alone on the woodlice (*Porcellio scaber*) exposed to 0.02%, 0.06%, 0.17%, 0.5% and 1.5% (w/w) virgin LDPE in Lufa 2.2 soil. Normality was tested with Kolmogorov-Smirnov tests, and homogeneity of the variances was verified with Levene tests. For data with parametric distribution and homoscedasticity, one-way ANOVA followed by Tukey tests was performed, while for data with non-parametric distribution or not achieving homoscedasticity, non-parametric Kruskal-Wallis tests followed by pairwise comparisons with the control was performed using Mann-Whitney U-tests.

**Parameter**	**ANOVA**		**Kruskal-Wallis**		Pairwise comparisons with the control **(Tukey or Mann-Whitney U-tests, see Methods)**									
	**F**	**p**	**$\chi 2$**	**p**	**0.02% w/w**		**0.06% w/w**		**0.17% w/w**		**0.5% w/w**		**1.5% w/w**	
					**U**	**p**	**U**	**p**	**U**	**p**	**U**	**p**	**U**	**p**
Survival	-	-	9.957	0.076	112.5	1.000	105	0.351	105	0.351	135	0.157	112.5	1.000
Feeding	-	-	18.074	0.003	58	2.889	81	0.461	115	0.434	34	0.032	45	0.027
Total haemocyte count	2.751	0.035	-	-	-	0.999	-	0.998	-	0.941	-	0.101	-	0.672
Haemocyte viability	1.169	0.346	-	-	-	0.697	-	0.477	-	0.730	-	0.245	-	0.742
Semigranulocytes	3.152	0.020	-	-	-	0.783	-	0.498	-	0.813	-	0.011	-	0.115
Granulocytes	4.167	0.005	-	-	-	0.867	-	0.997	-	0.984	-	0.017	-	0.038
Hyalinocytes	1.994	0.106	-	-	-	0.997	-	0.176	-	0.866	-	0.978	-	0.999

-, not applicable

Table S3. Collected test statistics on the effects of the recycled LDPE alone on the woodlice (*Porcellio scaber*) exposed to 0.02%, 0.06%, 0.17%, 0.5% and 1.5% (w/w) recycled LDPE in Lufa 2.2 soil. Normality was tested with Kolmogorov-Smirnov tests, and homogeneity of the variances was verified with Levene tests. For data with parametric distribution and homoscedasticity, one-way ANOVA followed by Tukey tests was performed, while for data with non-parametric distribution or not achieving homoscedasticity, non-parametric Kruskal-Wallis tests followed by pairwise comparisons with the control was performed using Mann-Whitney U-tests.

**Parameter**	**ANOVA**		**Kruskal-Wallis**		**Pairwise comparisons with the control (Tukey or Mann-Whitney U-tests, see Methods)**									
					**0.02% w/w**		**0.06% w/w**		**0.17% w/w**		**0.5% w/w**		**1.5% w/w**	
	**F**	**p**	**χ^2**	**p**	**U**	**p**	**U**	**p**	**U**	**p**	**U**	**p**	**U**	**p**
Survival	-	-	4.341	0.501	105	0.577	105	0.577	97.5	0.164	120	0.653	105	0.577
Feeding	-	-	3.382	0.641	112	0.320	101	0.645	86	0.612	71	0.724	88	0.903
Total haemocyte count	-	-	2.899	0.716	37	0.637	27	0.637	37	0.962	29	0.531	49	0.229
Haemocyte viability	1.772	0.138	-	-	-	0.999	-	0.998	-	0.583	-	0.998	-	0.915
Semigranulocytes	2.070	0.087	-	-	-	1.000	-	0.828	-	0.948	-	0.479	-	0.144
Granulocytes	0.905	0.486	-	-	-	1.000	-	0.823	-	1.000	-	0.872	-	0.806
Hyalinocytes	2.576	0.0392	-	-	-	0.998	-	1.000	-	0.441	-	0.793	-	0.202

-, not applicable

Table S4. TXRF-quantified concentrations of selected metals in *Daphnia magna* chronic reproduction assay treatments (100 mg recycled LDPE/L and untreated *D. magna* control). Metal concentrations are given as mg metal/L (AVG ± SD, n=2). LOQ – Limit of Quantification.

	***D. magna* exposure medium**	
**Metal**	**100 mg recycled LDPE/L**	**Control**
P	0.45 ± 0.06	0.46 ± 0.03
K	2.09 ± 0.30	2.57 ± 0.98
Ca	36.4 ± 2.20	36.7 ± 2.55
Ti	<LOQ	<LOQ
Fe	<LOQ	<LOQ
Cu	<LOQ	<LOQ
Zn	<LOQ	<LOQ
Pb	<LOQ	<LOQ

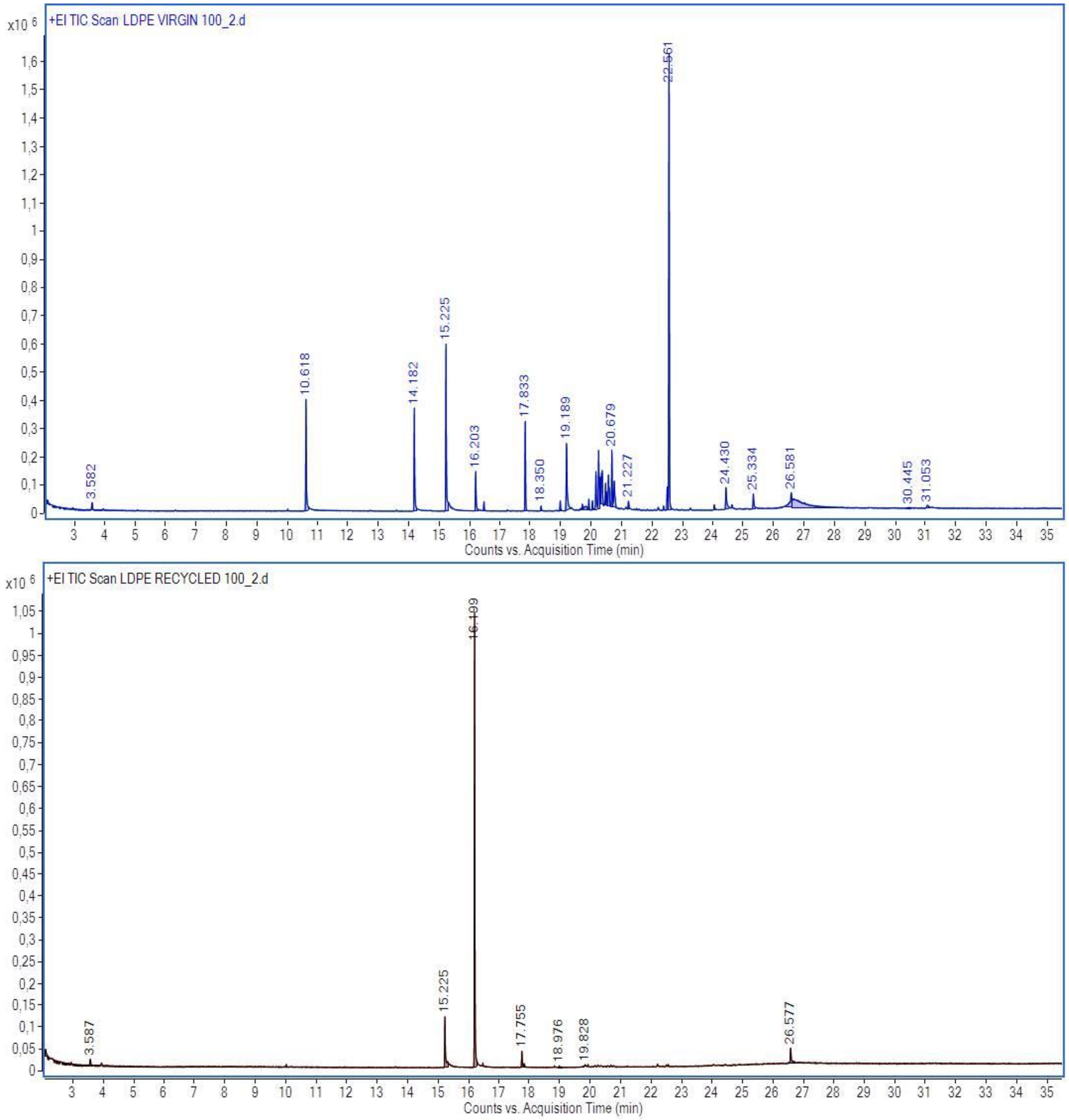

Figure S6. GC-MS chromatogram of virgin (top) and recycled (bottom) LDPE. Methanol extraction was used to prepare the samples.

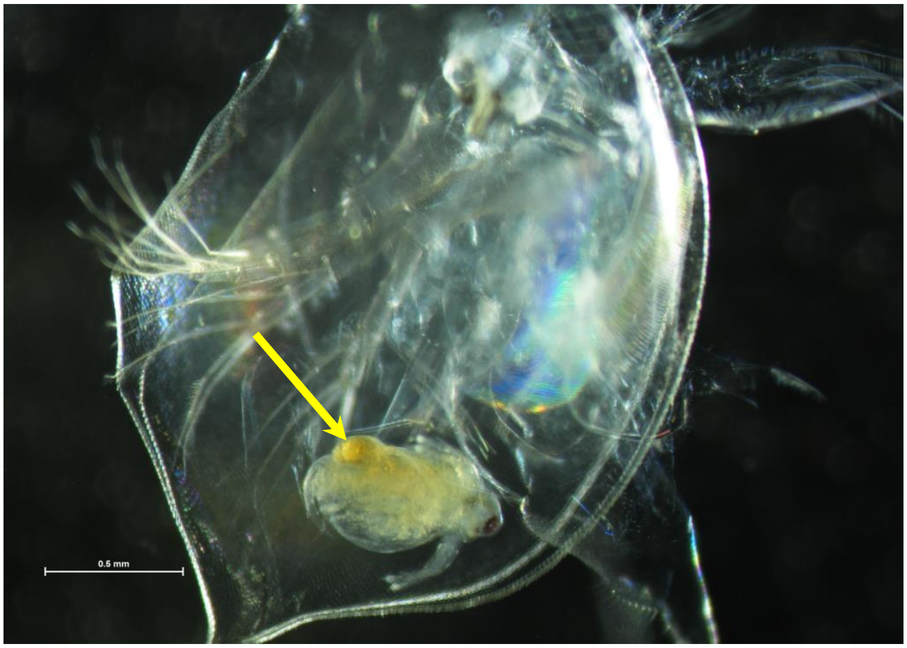

Figure S7. An embryo, retained in the shed carapace of *Daphnia magna* that had been exposed (18 days until this observation) to 100 mg/L of recycled LDPE. Yellow arrow indicates potential abnormalities of the embryo. Scale bar equals 0.5 mm.
